# A head with two tales: demystifying early spread of disease from the pancreatic head

**DOI:** 10.1007/s00261-024-04438-x

**Published:** 2024-06-21

**Authors:** Swetha Aribindi, Michael Oliphant, Janardhana Ponnatapura

**Affiliations:** Department of Diagnostic Radiology, Wake Forest University School of Medicine, 1, Medical Center Blvd, Winston Salem, NC 27157 USA

**Keywords:** Pancreas, Adenocarcinoma, Extraperitoneum, Lymphatic spread

## Abstract

The pancreas is a centrally located extraperitoneal organ within the anterior pararenal space. It is extensively connected to the extraperitoneal spaces by location and a network of mesenteries and ligaments. This provides interconnected avenues for vessels, lymphatics, and nerves to course through—as well as avenues for the spread of disease. The head of the pancreas results from the fusion of its ventral analog (anterior head) with its dorsal analog (posterior head). This differentiation provides two distinct pathways of spread of disease from the head of the pancreas. This communication will discuss the embryology, anatomy, and pathways of disease spread from the anterior and posterior pancreatic head. While any disease process can use these pathways, proven cases of adenocarcinoma of the pancreas are used for illustrations.

## Learning objectives

- Understand the embryology and anatomy of the pancreas.
- Understand the differences in early disease spread from the anterior and posterior head of the pancreas.
- Understanding how imaging helps in successfully localizing spread of disease from the pancreatic head.

## Embryology

During embryonic development, the primitive gut is suspended from the extraperitoneum by the primitive mesentery forming the ventral mesogastrium and the dorsal mesogastrium. The liver develops within the ventral mesogastrium part of which forms the gastrohepatic ligament (lesser omentum), and the hepatoduodenal ligament. The spleen and dorsal portions of the pancreas develop in the dorsal mesogastrium forming the splenorenal ligament, gastrosplenic ligament, the gastrocolic ligament, and greater omentum. The dorsal mesentery gives rise to the small intestine mesentery and mesocolon. These mesenteries and ligaments are identified by their contained vascular landmarks. These ligaments are interconnected to the anterior pararenal space in the region of the pancreas [1].

The pancreas is formed by two endodermal foregut diverticula, the ventral diverticulum and the dorsal diverticulum. The ventral diverticulum forms the ventral portion of the pancreatic head, and the dorsal diverticulum forms the dorsal portion of the pancreatic head [2]. Initially, the ventral and dorsal pancreatic buds are about 180° apart from each other. At around six to seven weeks of development, the gut rotates in the counterclockwise direction, moving the ventral pancreatic bud 180° toward the dorsal pancreatic bud [3]. The pancreatic buds fuse, with the ventral pancreatic bud forming the more posterior and caudally located posterior aspect of the pancreatic head and the dorsal bud forming the more anterior and cranially located portion of the pancreatic head, as well as the body and tail of the pancreas. The pancreatic duct from the dorsal bud fuses with the duct from ventral bud to form the main pancreatic duct (duct of Wirsing) which drains into the duodenum via the major papilla [1].

## Anatomy

The arteries and veins supplying the pancreas course within the ligaments and mesenteries surrounding the pancreas. Table 1 identifies these ligaments and mesenteries and their vascular landmarks.

**Table 1:** Ligaments and mesenteries associated with head of the pancreas

Peritoneal Ligament/Fold	Vascular landmark
Gastrohepatic ligament	Left gastric artery and vein
Hepatoduodenal ligament	Hepatic artery Portal vein Common bile duct
Splenorenal ligament Gastrosplenic ligament Gastrocolic ligament Greater omentum	Splenic artery and vein Left gastroepiploic artery and vein Right and left gastroepiploic arteries and veins Epiploic arteries and veins
Transverse mesocolon Dorsal mesoduodenum	Middle colic artery and vein Gastrocolic trunk Gastroduodenal artery
Root of small bowel mesentery	Superior mesenteric artery Jejunal arteries and veins

The anterior head of the pancreas is connected to adjacent structures by the gastrohepatic ligament, hepatoduodenal ligament, the dorsal mesoduodenum, and the transverse mesocolon.

The posterior head of the pancreas is connected to adjacent structures by its dorsal mesentery and the root of the small intestine mesentery.

## Arterial supply

**Fig. 1:**
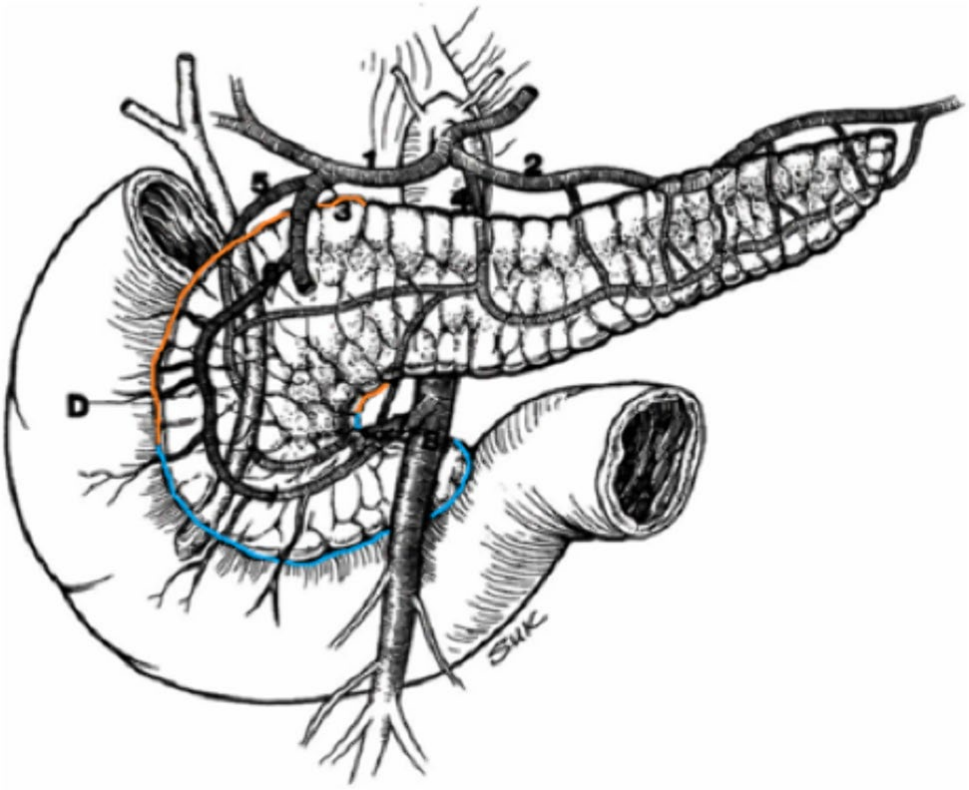
The arterial supply to the anterior pancreatic head (outlined in orange) and posterior pancreatic head (outlined in blue). 1 = common hepatic artery (CHA); 2 = splenic artery; 3 = gastroduodenal artery (GDA); 4 = dorsal pancreatic artery; 5 = posterior superior pancreaticoduodenal artery (PSPDA); 6 = anterior PSDA; 7 = inferior pancreaticoduodenal artery (IPDA); 8 = superior mesenteric artery (SMA); D = duodenum. Reproduced with minor editing from “Meyers, M. A., Charnsangavej, C., Oliphant, M. (2011). *Meyers’ dynamic radiology of the abdomen: normal and pathologic anatomy*. New York, NY: Springer” with permission from Springer

The anterior head of the pancreas is primarily supplied by the common hepatic artery (CHA), a branch of the celiac artery (Fig. 1). The CHA gives rise to the gastroduodenal artery (GDA), which then branches into the posterosuperior pancreaticoduodenal artery (PSPDA) and anterosuperior pancreaticoduodenal artery (ASPDA). The terminal branch of the GDA is the right gastroepiploic artery which courses in the gastrocolic ligament along the greater curvature of the stomach. The dorsal pancreatic artery arising from the celiac artery or the splenic artery supplies the anterior head medially [1].

The posterior pancreatic head is primarily supplied by the inferior pancreaticoduodenal artery (IPDA), which arises from the superior mesenteric artery (SMA) or the proximal jejunal artery branching off the SMA (Fig. 1). The IPDA then branches to the AIPDA and PIPDA which anastomose with the ASPDA and the PSPDA [1].

## Venous drainage

The anterior head of the pancreas is drained by the anterosuperior and posterosuperior pancreaticoduodenal veins (ASPDV and PSPDV respectively). The ASPDV drains into the gastrocolic trunk (located in the gastrocolic ligament), which then drains into the superior mesenteric vein (SMV) anteriorly. The PSPDV drains into the inferior surface of the portal vein (PV), near the confluence of the SMV and splenic vein [1]. The posterior pancreatic head is drained by the inferior pancreaticoduodenal vein, which drains into the proximal jejunal vein. The proximal jejunal vein drains into the posterior aspect of the SMV [1].

## Lymphatic drainage

The lymphatic drainage of the pancreatic head follows the arterial supply of the pancreatic head [1].

The anterior pancreatic head drains its lymphatics to the anterior and posterior pancreaticoduodenal nodes. Lymph nodes in this pathway include the infrapyloric nodes. Lymph then drains along the GDA to the proper hepatic artery, the CHA, and then to the periportal lymph nodes. Drainage is then along the celiac artery to the celiac node [1, 2].

The posterior pancreatic head is drained along the inferior anterior and posterior arteries to the corresponding inferior pancreaticoduodenal nodes. Drainage continues into the SMA node or less frequently to the proximal jejunal nodes [1, 2].

## Nervous supply

Innervation to the pancreatic head is supplied mainly by the celiac plexus and the superior mesenteric plexus. These nerve plexuses and their derivatives are closely associated with the arterial supply to the pancreatic head. The nerves that derive from the celiac plexus are divided into the anterior and posterior hepatic plexus [1, 4].

The anterior hepatic plexus runs along the CHA, proper hepatic artery, GDA, and pancreaticoduodenal artery to innervate the ventral aspect of the anterior pancreatic head. The posterior hepatic plexus originates from the right celiac plexus/ganglion and runs along the PV and common bile duct to innervate the dorsal aspect of the anterior pancreatic head [1, 5, 6].

The nerves that derive from the superior mesenteric plexus are divided into the direct pathway and the accompanying pathway. The nerves in the direct pathway directly enter the posterior pancreatic head from the superior mesenteric plexus, while the nerves in the accompanying pathway follow along the path of the inferior pancreaticoduodenal artery into the posterior pancreatic head [6].

## Pattern of disease spread from the anterior head

### Lymphatic spread

**Fig. 2:**
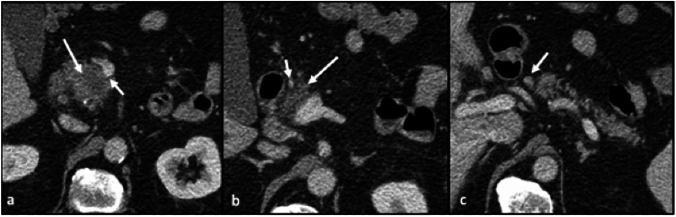
Axial CT sections with contrast in the portal venous phase. **a** Hypoenhancing mass in the anterior pancreatic head (long arrow), with slight flattening and deformation of the SMV (short arrow). **b** Disease infiltrates superiorly into the dorsal mesoduodenum (long arrow), demarcated by the gastroduodenal artery (arrow). **c** Enlarged infrapyloric node, indicating early involvement of disease spread (arrow)

The lymphatic spread from the anterior pancreatic head is a complex network, but primarily involves a few key nodes: the celiac node (a principal node for spread of disease from the anterior pancreatic head), infrapyloric nodes, and periportal nodes (Fig. 2).

### Mesenteric spread

**Fig. 3:**
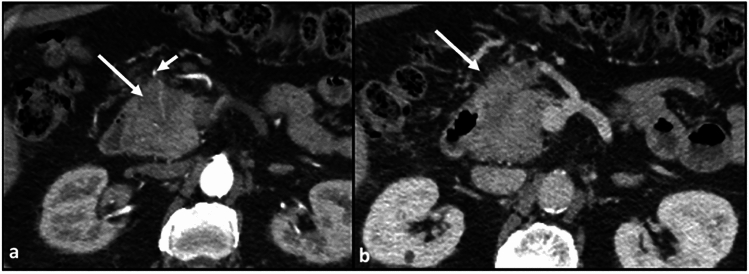
Axial CT sections with contrast in the arterial phase (**a**) demonstrates heterogenous hypodense mass in the anterior pancreatic head (long arrow), with disease infiltrating adjacent structures and encasing the gastroduodenal artery (short arrow). Axial CT section with contrast in the portal venous phase (**b**) demonstrates infiltration of disease through the dorsal mesoduodenum into the duodenum, with early infiltration into the transverse mesocolon as well (arrow)

Mesenteric spread from the anterior pancreatic head involves the following: the dorsal mesoduodenum (spread along GDA), the hepatoduodenal ligament (spread along the hepatic artery), the gastrohepatic ligament (spread along the left gastric artery), the gastrocolic ligament, (spread along the right gastroepiploic artery), and the transverse mesocolon (spread along the right and middle colic arteries) (Fig. 3).

### Direct spread

**Fig. 4:**
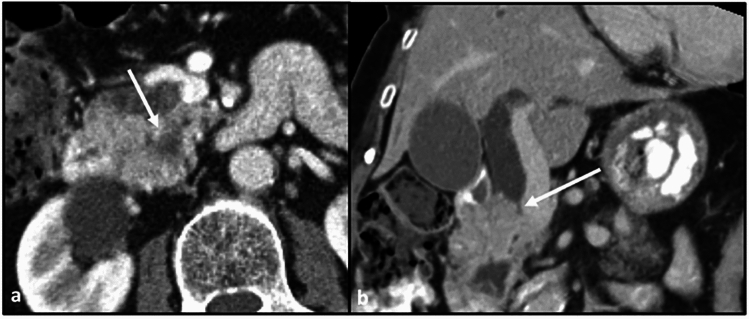
Axial CT sections with portal venous phase contrast. **a** Ill-defined hypodense mass in the anterior pancreatic head (arrow). **b** Obstruction of the common bile duct above the level of the ampulla (arrow)

Direct spread of disease from the anterior pancreatic head involves adjacent structures including the common bile duct above the level of the ampulla, the proximal aspect of the 2nd segment of the duodenum, the distal stomach, and the transverse mesocolon, through which disease can reach the transverse colon and hepatic flexure (Figs. 3 and 4).

### Perineural spread

**Fig. 5:**
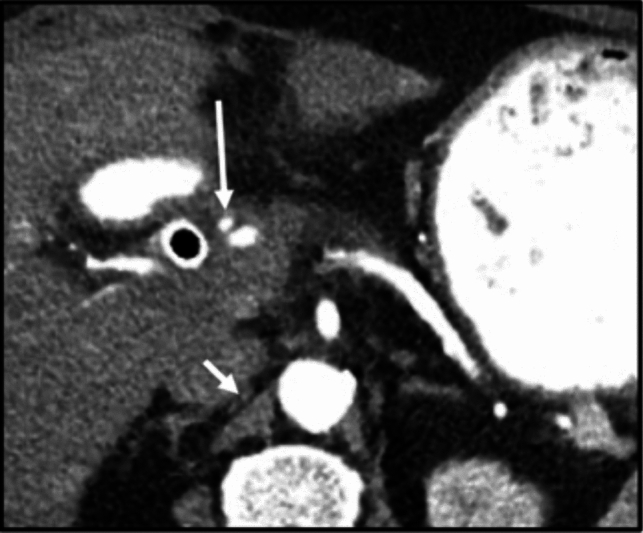
Axial CT section with arterial phase contrast. Ill-defined mass in the anterior pancreatic head, which encases the gastroduodenal artery (long arrow). Disease infiltrates close to the right celiac ganglion (short arrow)

Perineural spread from the anterior pancreatic head involves the right celiac ganglion (Fig. 5).

## Pattern of disease spread from the posterior head

### Lymphatic spread

**Fig. 6:**
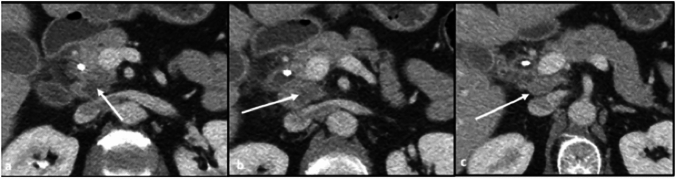
Axial CT sections with portal venous phase contrast. **a, b** Hypoenhancing mass in the posterior pancreatic head/uncincate process (arrows). **c** Abnormally enlarged, hypoenhancing retropancreatic node, indicating malignant involvement (arrow)

Lymphatic spread from the posterior pancreatic head involves the superior mesenteric node, the inferior pancreaticoduodenal nodes, and aortocaval nodes to the level of the L2 vertebral body (Fig. 6), and occasionally the proximal jejunal nodes.

### Mesenteric spread

**Fig. 7:**
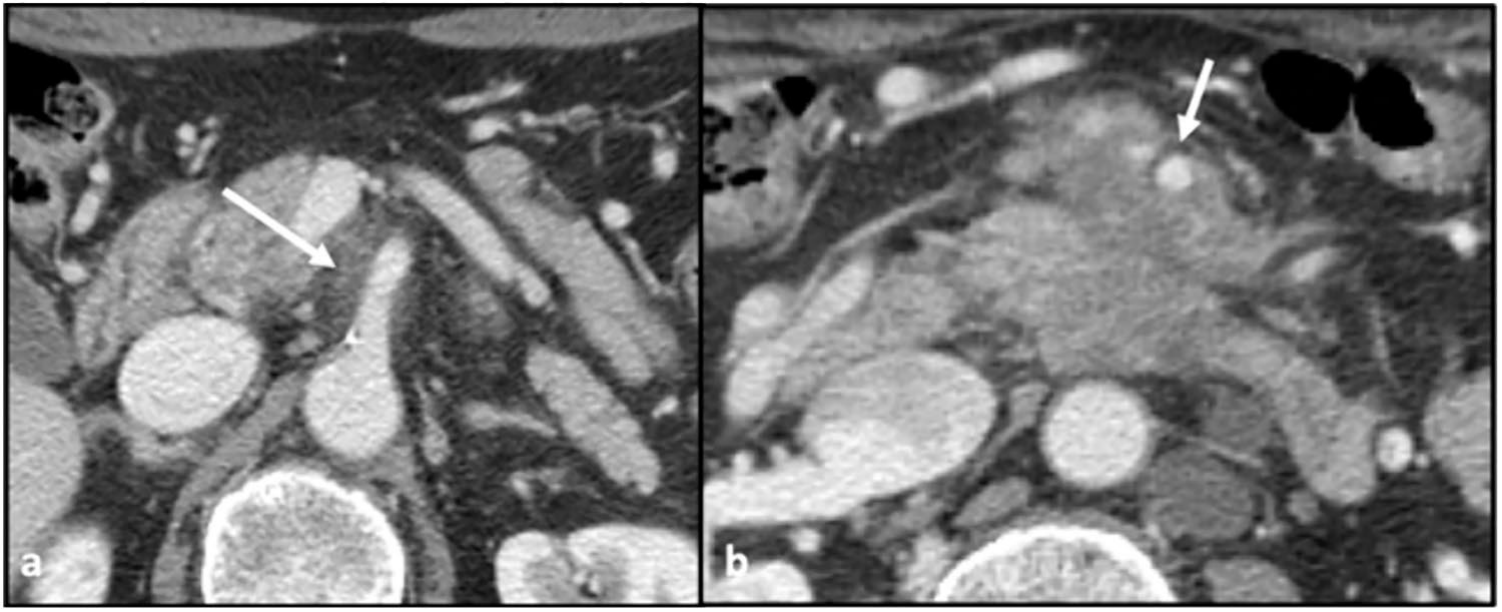
Axial CT section with portal venous phase contrast. **a** Infiltrative disease spread along the base of the small bowel mesentery, delineated by the superior mesenteric artery (arrow). **b** Disease spread along the more distal aspect of the superior mesenteric artery, in the jejunal mesentery (arrow)

**Fig. 8:**
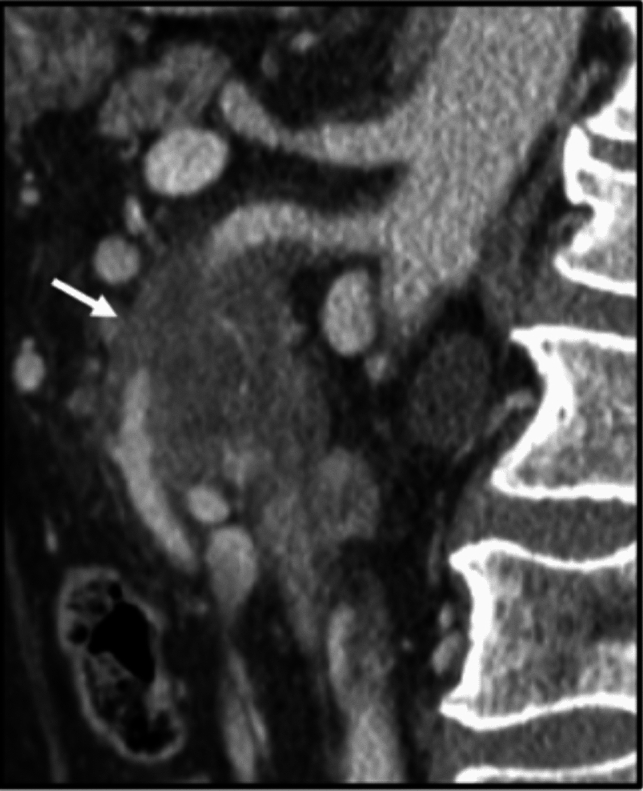
Sagittal CT section with portal venous phase contrast. Hypoenhancing tumor encases the superior mesenteric artery (arrow)

Mesenteric spread from the posterior pancreatic head involves the base of the small intestine mesentery (demarcated by the SMA, can involve replaced right hepatic arteries originating off the SMA, the mesentery around the inferior pancreaticoduodenal artery, and the jejunal mesentery (jejunal arteries) (Figs. 7 and 8).

### Direct spread

**Fig. 9:**
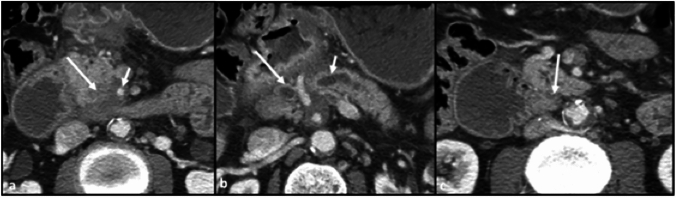
Axial CT sections with contrast in the portal venous phase. **a** Ill-defined, hypodense mass in the posterior pancreatic head (long arrow), which encases the superior mesenteric artery (short arrow). **b** Dilatation of the common bile duct (long arrow) and the pancreatic duct (short arrow), indicating obstruction at the level of the ampulla. **c** Infiltration of disease into the 3rd segment of the duodenum (arrow)

Direct spread from the posterior pancreatic head involves adjacent structures such as the distal aspect of the common bile duct (at the level of the ampulla) and the distal duodenum (such as the 3rd segment and the inferior genu) (Fig. 9).

### Perineural spread

**Fig. 10:**
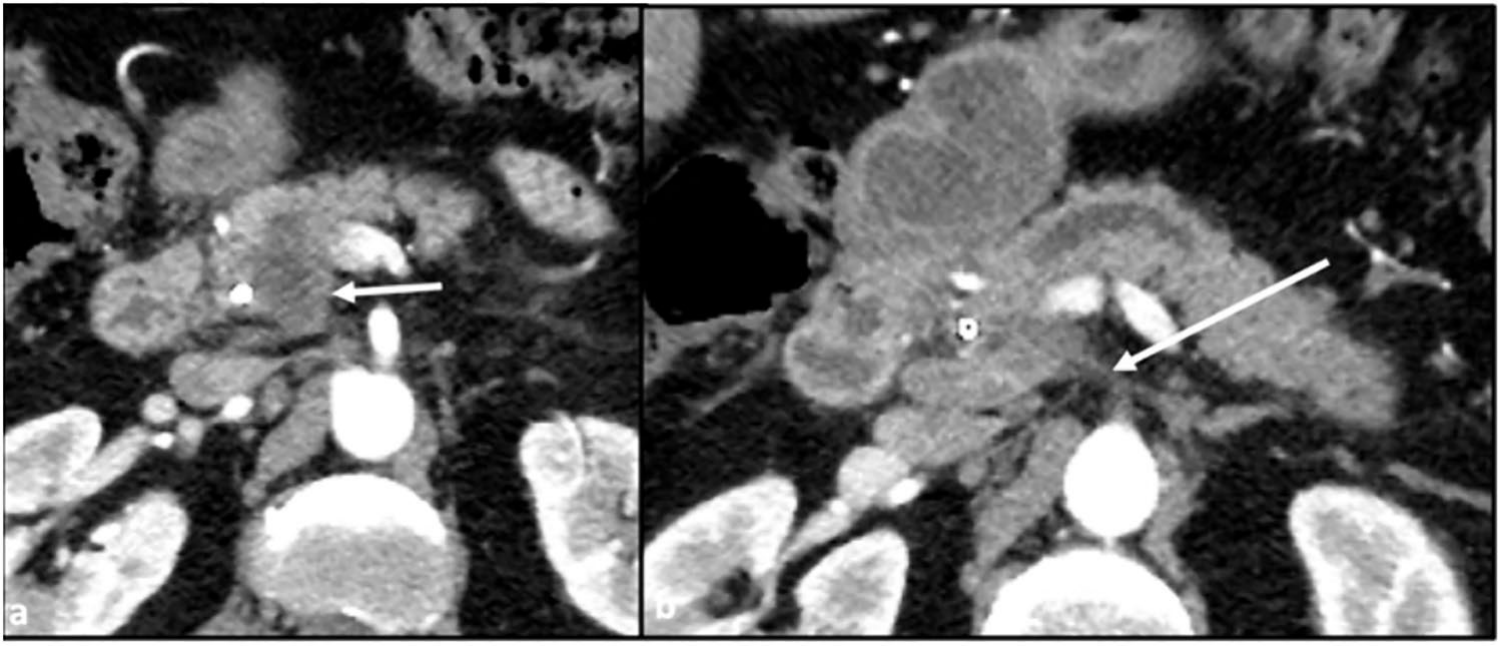
Axial CT sections with contrast in the arterial phase. **a** Hypodense mass in the posterior pancreatic head (arrow). **b** Early spread of disease into the superior mesenteric plexus (arrow)

Perineural spread from the posterior pancreatic head involves the superior mesenteric plexus/ganglion around the SMA (Fig. 10).

## Conclusion

The purpose of this article is to describe the two distinct spread patterns of disease from pancreatic head. It is important to start with a brief discussion of the embryology as it forms the basis of understanding the distinct spread patterns. The anterior and posterior areas of the pancreatic head develop separately and contain separate vascular, lymphatic, and nerve supply. These structures form the pathways for disease spread and are summarized in Table 2. Any disease process, including inflammation, infection, and tumor, can use these pathways. Knowledge of these spread patterns allows for a nuanced search pattern for detecting spread. Detailed report of disease spread is essential for staging and treatment planning. Additionally, disease can present at a distance from its origin and knowledge of the spread patterns can lead to its origin source.

**Table 2:** Different pathways for disease spread from anterior head and posterior head of the pancreas

Type of spread	Anterior head	Posterior head
Lymphatic spread	• Celiac node (principal node) • Infrapyloric node • Periportal node	• SMA node (principal node) • Retropancreatic node • Aortovenous nodes to the level of L2
Perineural spread	Right celiac ganglion	Superior mesenteric ganglion
Mesenteric spread	• Dorsal mesoduodenum (along the gastroduodenal artery) • Ventral mesogastrium (gastrohepatic ligament and hepatoduodenal ligament)	• Dorsal mesentery of the pancreas (along the inferior pancreaticoduodenal artery) • Jejunal mesentery (along the SMA base and jejunal arteries)
Direct spread	• Obstructs the CBD above the ampulla • Proximal duodenum (2nd segment) • Distal stomach • Transverse mesocolon into the transverse colon (hepatic flexure)	• Obstructs the CBD at the level of the ampulla • Distal duodenum (3rd segment, inferior genu)
